# A rich diversity of opercle bone shape among teleost fishes

**DOI:** 10.1371/journal.pone.0188888

**Published:** 2017-12-27

**Authors:** Charles B. Kimmel, Clayton M. Small, Matthew L. Knope

**Affiliations:** 1 Institute of Neuroscience, University of Oregon, Eugene, Oregon, United States of America; 2 Institute of Ecology and Evolution, University of Oregon, Eugene, Oregon, United States of America; 3 Department of Biology, University of Hawaii at Hilo, Hilo, Hawaii, United States of America; Laboratoire de Biologie du Développement de Villefranche-sur-Mer, FRANCE

## Abstract

The opercle is a prominent craniofacial bone supporting the gill cover in all bony fish and has been the subject of morphological, developmental, and genetic investigation. We surveyed the shapes of this bone among 110 families spanning the teleost tree and examined its pattern of occupancy in a principal component-based morphospace. Contrasting with expectations from the literature that suggest the local morphospace would be only sparsely occupied, we find primarily dense, broad filling of the morphological landscape, indicating rich diversity. Phylomorphospace plots suggest that dynamic evolution underlies the observed spatial patterning. Evolutionary transits through the morphospaces are sometimes long, and occur in a variety of directions. The trajectories seem to represent both evolutionary divergences and convergences, the latter supported by *convevol* analysis. We suggest that that this pattern of occupancy reflects the various adaptations of different groups of fishes, seemingly paralleling their diverse marine and freshwater ecologies and life histories. Opercle shape evolution within the acanthomorphs, spiny ray-finned fishes, appears to have been especially dynamic.

## Introduction

Understanding the patterns of morphological diversifications among organisms constitutes a central goal of evolutionary biology. These patterns can be quantified using multivariate approaches such as geometric morphometrics [1,2] and visualized as plots on spaces of reduced dimensionality. Such spaces are known as morphospaces, and the occupancy within the space may be termed the morphospace landscape (i.e., denoted by the collective of datapoints of the scatterplot). Of special interest in our study is that it has been understood from previous theoretical and empirical studies that a morphospace landscape generally is not uniform in appearance, but sparsely occupied and clumpy [3–10]. The clustering is claimed to be independent of scale–“from populations to the highest taxonomic categories” [8], which if true would seem remarkable. What kind of evolutionary process(es) could result in such a broadly shared evolutionary pattern?

Within a morphospace framework, evolution can be thought of as a suite of processes—adaptive or otherwise—that modify landscape features. The change in position between an ancestor and a descendant would be visualized as a trajectory, a specific pathway, through the morphospace. This phylomorphospace approach, that we use here to visualize these trajectories [11], requires not only being able to measure morphologies (e.g. of extant forms or well-preserved fossils) such that the morphologies can be plotted, but also knowing the phylogenetic relationships of the forms in question. With a phylogeny in hand the positions of ancestors, essentially unavailable for direct measurement, even if there is something of a fossil record, can be estimated by computation (e.g. by squared change parsimony; [12]). These computed ancestral positions in the landscape are then compared with the measured positions of the descendent set. The phylomorphospace, showing the trajectories, provides a way to understand trait evolution.

In this study we examine the shapes, and their evolution, of a single skull bone, the opercle (OP), among an extremely broad group of organisms, the teleost fishes. Teleosts, by far the most diverse group of vertebrates, are classified into 66 orders (sensu [13]), more than 500 families, and more than 30,000 species. The morphological differences among the head skeletons of these species can be quite dramatic [14]. Our interest in examining just a single bone is because it is possible, as we have shown for zebrafish, to consider how it is shaped in considerable detail, at the level of cell biology and developmental genetics [15–18]. This means that the evolutionary patterns we observe, here at the macroevolutionary scale, may eventually be understandable at a very refined and mechanistic level through complementary approaches from comparative and developmental biology. The OP, providing the principal support of the gill cover, functions critically in respiration and mouth opening [19,20]. Hence, we can contemplate possible ecological determinants of morphological change. Also motivating our current study was our exploration of evolutionary shape change within threespine stickleback. We found the local morphospace representing the oceanic-freshwater divergence of a number separate populations across the world, to be rather densely occupied (S1 Fig; [21]). Does this microevolutionary pattern have a counterpart in macroevolution?

Here we extend the stickleback work by examining opercle disparities among teleost families, in contrast to the within-species stickleback data. Earlier studies have broadly examined single kinds of bones broadly among fish species, but with different motives than ours. For example, McAllister examined branchiostegal rays in an attempt to use them as systematic characters (i.e., to determine phylogenetic relationships) [22], and Arratia and Schultze focused on homology of a hyoid tendon bone, the urohyal, describing it as a unique feature of teleosts [23]. We choose the family taxonomic level for this analysis because, as we show below, the shape differences among the families are prominent, and as expected, considerably more so than within species, or even within families as revealed by recent work examining Cichlidae and Ariidae [24,25]. To look for directional channeling of evolutionary shape change, especially evolutionary convergences, we use a phylomorphospace approach. We find mostly dense and fairly unclustered morphospace distributions. The phylomorphospace findings suggest the presence of marked evolutionary excursions through space, including both divergences and convergences. Especially considering recent work describing “explosive” responses of acanthomorphs to fill in a region of morphospace left barren by the end Cretaceous mass-extinction event [26,27], our findings lend support to ecology as being a major determinant of morphospace patterning, again matching interpretations of the possible cause of stickleback microevolution.

## Materials and methods

### Ethics

The study was approved by the University of Oregon IACUC. No living animals were utilized or euthanized specifically for the purpose of this study: The preserved fish (or just their heads) were obtained as by-products from other projects, including gifts from individuals or gifts or loans from scientific collections (US Marine Fishery, NOAA; Oregon State University Ichthyology Collection) and otherwise (e.g. sport fishery, hobby tropical fish suppliers).

### Specimen collecting and preparation

Our survey is based on species from 110 families, therefore representing about a fifth of the approximately 500 families of Actinopterygians, ray-finned bony fishes. All but three families are teleosts. The species and the families they represent are broadly distributed across the phylogenetic tree, however, virtually no deep-sea groups are represented. 103 of our families are included in the tree of Betancur-R et al. [13], which we used to construct the phylogeny for our analyses. Dissections, photographic imaging and morphometric analyses were initially carried out with 237 preparations representing 202 species: eventually this dataset was reduced to single samples for each species by averaging shape coordinates in cases where we had multiple individuals from the same species. In the interest of equal taxon sampling, for families for which we had multiple representatives, we culled to a single ‘exemplar’ species for each family; the final list is reported in S1 Table. Because quantitative bone shape studies have not previously been done for the great majority of species studied here, we have no way of knowing whether the chosen examples are truly representative of the groups. Examples of the within-family variation as compared with among family variation for our study set are shown in S2 Fig.

**Fig 1:**
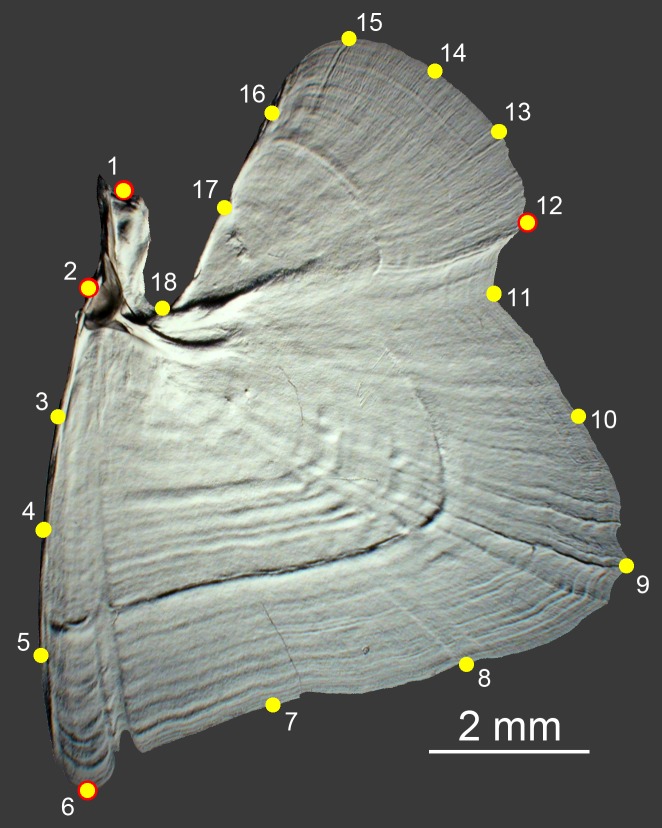
Landmarking the OP. The distribution along the bone edges of four landmarks (LM, yellow, red border) and 14 semilandmarks (yellow) used in this study, in a smelt (*Osmerus mordax*, Osmeridae). Developmental studies in zebrafish, stickleback, cory catfish, and *Oncorhynchus* species inform the positions of the landmarks, those that appear to correspond among all of the species examined [15,29,30], and unpublished: These landmarks include LM1 (joint-associated), LM2 (socket of the ball-and-socket joint the OP makes with the hyomandibula), LM6 (ventral apex) and LM12 (posterior apex, associated with a prominent strut that Gregory termed a tract of folded trabeculae [14] traversing the bone from the joint region). The opercular dilator muscle connects to the OP at LM1. The opercular levator muscles connect between LM1 and LM12. The prominent curvature at LM9 is also shared among many species, including zebrafish (termed ‘c’ in [15]. The semilandmarks were placed either at constant intervals along the appropriate segment of the edge, or to capture elaborations of the edge in particular species. There is no basis currently for supposing that any of the semlandmarks capture homologous locations among the families, even though some them appear consistent among species within families.

We dissected the OP from the skull, noting positional relationships with neighboring bones and the locations of muscle attachments to aid in eventual identification of landmark positions. Dissection, allowing the individual bones to be examined in detail in their entirety, is by far our preferred method for analyzing bone morphology and landmarking. Dissection was sometimes aided by digestion of soft tissues with trypsin and cleaning by dermestid beetles. We also preferred to avoid formaldehyde (formalin) preserved material, which toughens soft tissue, including ligaments, and makes dissections of the sometimes very delicately mineralized bones more difficult. For this reason, and because dissection is destructive we mostly avoided using museum specimens. We obtained most specimens as fresh frozen preparations. Some specimens were preserved in 70% or 95% ethanol. Smaller species were briefly fixed or postfixed (generally for 2–4 hours) with 2 or 4% neutral formaldehyde, and then double-stained with Alcian blue–Alizarin Red, following a procedure that minimizes mineral loss (and hence preserves Alizarin Red staining) during preparation [28]. Following staining and clearing, the bones were separated by dissection in a solution of 50% glycerol and 0.01% KOH (final concentrations). Whether the bones were stained or not, we did not allow drying throughout the procedures: Wet preparations are preferable for revealing subtleties of bone structure which aids in landmarking the bones (Fig 1).

The OPs, usually from both the left and right sides of individual skulls, and often along with other selected craniofacial bones, were photographed, including both medial and lateral views. Digital images were taken with a Leica stereomicroscope equipped for epifluorescence for the smaller preparations and with a Nikon macro setup for the larger ones. In either case we used an incident light source, offset for the bright field images to provide relief, which aids in morphological study. Comparisons of the left-right morphologies of the bones themselves, as well as the images, provided a check for abnormalities that might arise as developmental accidents or due to injury. In fact, we essentially never encountered major left-right differences. A single OP from each specimen was selected for morphometric analysis.

### Morphometrics and data analyses

The OP, supporting the gill cover (operculum) is quite flat, such that 2D rather than 3D morphometric analyses are appropriate for shape analyses. Eighteen landmarks were placed around the edge of the OP for geometric morphometric analyses (Fig 1, [31,32]). The legend to Fig 1 provides detail concerning landmark placement. Landmark positions were marked on the digital images in Photoshop, and then digitized using tps Dig, version 2.12 [33]. The digitized raw datasets were moved to MorphoJ [32] for Procrustes transformation, which removes size and orientation from the bone shape analysis, and the resulting shape variables were ordinated and examined by principal component analysis (PCA). Because the first two principal components explained more than 50% of the total shape variation, along with a precipitous drop in variation explained by the remaining PCs, we based subsequent analyses on the PC1-PC2 morphospace. PC scores were transferred to JMP (SAS Institute Inc., Cary, NC, v. 8) for examining scatter plots including nonparametric density landscapes. The PC scores and time-calibrated phylogeny pruned from a massive phylogenetic tree of the teleosts [13] were then transferred to Mesquite [34] for visualization of phylomorphospace [11]. We also used morphol.disparity and compare.evol.rates functions from the R package geomorph (version 3.0.0) [35] for comparing morphological disparities [31] and evolutionary rates of divergence [36,37] in a phylogenetic context. We tested for phylogenetic signal using “K” metrics [38], determining a multivariate version of K from the Procrustes coordinates [39], as implemented in geomorph. Using the R package *GEIGER* [40,41], we evaluated the fit to our PC data (the first two principal components) of four models of phenotypic evolution: “early burst” [42,6,43], Brownian motion, white noise, and Ornstein-Uhlenbeck. We also tested for phenotypic convergence in the PC1-PC2 phylomorphospace using the R package *convevol* [44] (see [45] for a working definition of convergence, as used in this study). Specifically, we tested for convergence in groups of taxa associated with density features of the landscape, including high- (“peaks”) and low- (“valleys”) density regions. Hypothesis tests for convergence, using the metrics described in Stayton [44], were carried out in *convevol* via 1000 Brownian motion simulations per test.

## Results

### Diverse opercle morphologies

**Fig 2:**
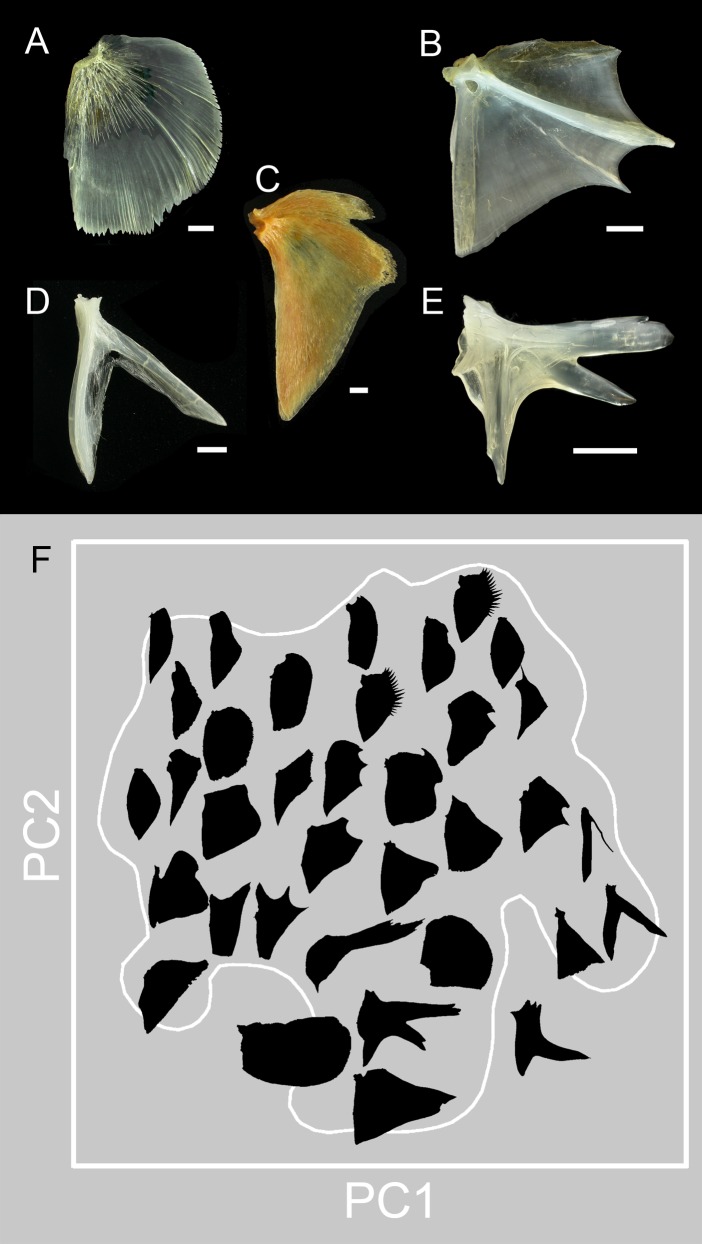
Opercles (OPs) show exceptional morphological diversity across species and families. **A** Salmon, *Oncorhynchus gorbuscha*, family Salmonidae, **B** sand bass, *Paralabrax nebulifer*, Serranidae, **C** reineta, *Brama australis*, Bramidae, **D** batfish, *Dibranchus atlanticus*, Ogcocephalidae, **E** toadfish, *Opsanus tau*, Batrachoididae. All of these examples show teleosts, and all but A are spiny-ray finned neoteleosts, acanthomorphs. Scale bars: 5 mm. **F** Principal component ordination arranges the OPs in a morphospace according to their shapes. The presentation emphasizes the exceptional variation we encountered in this survey. Here the bones, presented as silhouettes, are stripped of all of their distinguishing features (the location of the joint socket, presence of struts crossing the bone, outgrowth incremental banding pattern, density of Alizarin Red staining) except their shapes. We selected the examples to show the spread and mapping across the PC1 by PC2 morphospace. The white outline surrounding the occupied region of the space, the ‘landscape’, was made from nonparametric density contours. See text and Fig 3).

In agreement with classical work [14], we find prominent shape variation of the OP, the principal bone of the gill cover (operculum) of ray-finned fishes (Actinopterygii) (Fig 2). The bone is usually broad and sheet-like in planar view, but there are evident exceptions, e.g., Fig 2D. Some OPs exhibit prominent protrusions serving as attachment points to musculature or other skull elements, or apparently serving as defensive spines (e.g., Fig 2B). In all of our samples, the OP is flattened within the plane of the operculum. The socket of the ball-and-socket joint the OP makes with the hyomandibula (shown to the upper left for each example in Fig 2) is invariably present and appears conservative in structure. Other than the flattening and joint socket, we see marked diversity of opercle form. We note that shape deformations do not appear to be at all uniform across the bone edges. Spines, if present, generally adorn the posterior and ventral bone edges, sensible locations for protection since they are present over the region of opening of the gill chamber. Interestingly, a straight stretch of the anterior-ventral edge of the OP (between landmarks 2 and 6; Fig 1) seems highly conservative in shape among our samples (see Discussion), with a few exceptions (e.g. Fig 2E).

### Features of morphospace occupancy

**Fig 3:**
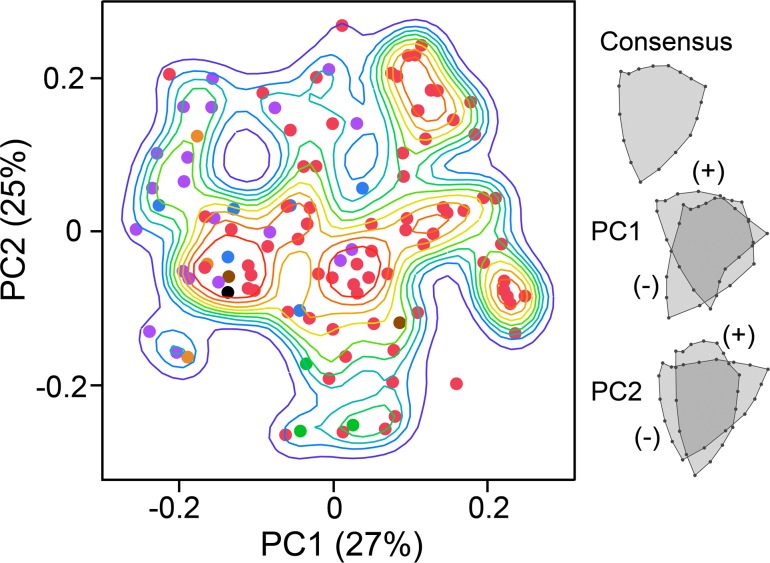
Principal component-based morphospace occupancy. PCA reveals that most of the bone shapes densely occupy a single large region of the space, here constructed from the two leading eigenvectors, PC1 and PC2, derived from landmark analysis. The percentages within the parentheses give variances explained. The accompanying overlay configurations show shape changes associated with the PC axes. PC1 captures details of the outlines: forked OPs toward high positive PC1 values and smooth configurations, often with prominent dorsal regions (upper in the figure) toward negative PC1 values. In contrast PC2 separates bones that are thin along the anterior-posterior axis (positive PC2 or much broader along this axis (negative PC2. The data points of the scatter plot show single exemplar species from each of the families across the phylogeny in Fig 4, with matching colors between the points here and the branches of the tree. The lines included with the scatterplot show nonparametric density contours. Heat map colored contours are according to occupancy density, with warm colors indicating density peaks. Morphospaces using other PCs (e.g. PC3 by PC4) also reveal dense occupancies.

A PCA-derived morphospace plot reveals a landscape that mostly looks densely occupied. Although some clustering is evident among the samples, and thus their distribution is not at all even, the landscape is not notably clumpy (Fig 3). To provide a check that this filled in pattern is not due to some technical artifact, we used an independent method of shape analysis of our samples, based on outlines rather than landmarks, and obtained a similar dense occupancy pattern to that in Fig 3, including a largely matching up of the spreads of the individual samples across the landscapes (S2A and S2B Fig). Similarly, lack of prominent clumping does not depend on our examining only a single species for each family. We also examined a morphospace in which some of the families are represented by multiple species (S2C Fig). This multiple species morphospace includes about twice as many data points and accordingly is more filled in (compare S2A and S2C Fig), but its pattern of distribution of points is otherwise reasonably similar to the single species one. The unequal taxon sampling present for the multiple species analysis might be expected to increase clustering, if anything.

**Fig 4:**
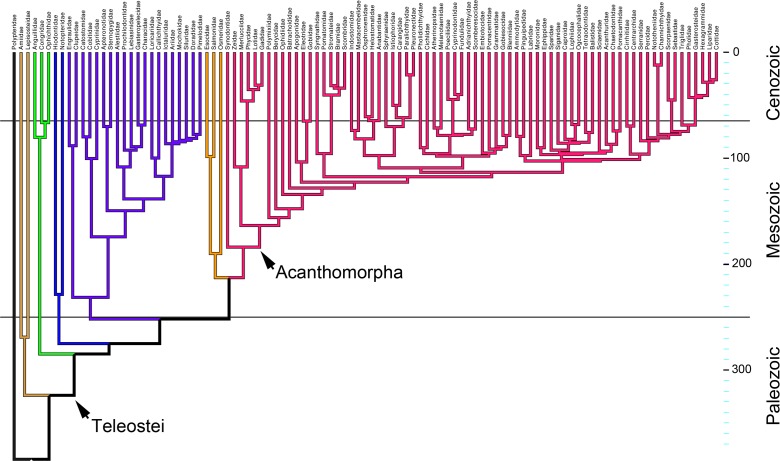
The time-tree showing families analyzed in this study and their times of origin. The tree was pruned from an extensive species-level time tree provided to us by Richard Betancur-R, using data from [13]. Branches along the tree indicate families. Color coding shows clades across the tree, and matches Fig 3.

As pointed out above, the distribution of data points in Fig 3 is not completely uniform, perhaps due in part to taxon sampling, but it also could have biological meaning–i.e., reflecting a regionally rugged morphological landscape, with multiple peaks and valleys. The presentation in Fig 3 conveys these rugged regions by including contour lines that show the nonparametric densities of local space occupancy. We used color coding of the data points to see, at large phylogenetic scale, how families broadly related to one another are distributed within these occupancy landscapes. The colors of the points in Fig 3 correspond to clades across the phylogenetic tree in Fig 4. For example, red indicates the neoteleosts, the largest teleost clade, arising deep within the teleosts. In our dataset, all of the neoteleost families except the basal-most one, Synodontidae, are spiny ray finned fishes, acanthomorphs, and they are mostly located on the right side of the landscape–toward high PC1. In contrast, most nonacanthomorph groups are present to the left, especially the upper left (low PC1, high PC2). Except for the ‘far right’ which is exclusively red, mixing among the groups represented by the various colors is considerable. The three families color-coded green, and representing the most basal teleost clade, are located toward the bottom of the landscape (low PC2), and share this location with acanthomorphs. The acanthomorph lineages outnumber the others, which partly accounts for their broader distribution. However, particularly the exclusive occupancy of the right side of the landscape (Fig 3, high PC1 values) by acanthomorphs strongly suggests that among the sampled taxa, their OP morphologies are rather more diverse than in the nonacanthomorphs examined. In agreement, *geomorph* tests using the morphol.disparity and compar.evol.rates functions, respectively, suggest that acanthomorph OP morphological disparity, estimated as Procrustes variance [31] is significantly higher than that of the nonacanthomorphs included in this study (1.52 times, p = 0.002; Table 1) and, further, that acanthomorph OP shapes have evolved significantly more rapidly than OPs of the nonacanthomorphs (2.33 times, p = 0.001; Table 1, [36,37]. We measured the tendency for related taxa to resemble one another, known as phylogenetic signal [38], using a multivariate version of Blomberg’s K [39]. We found K to be significant and relatively low, 0.53 (p = 0.0001, determined from permutation analysis). We can interpret low phylogenetic signal to be consistent with our visual impression of scattering among lineages, for scattering would reduce phylogenetic signal.

**Table 1:** Morphological disparities and morphological divergence rates of the OP in acanthomorphs and nonacanthomorphs.

Group	n	Disparity^1^	Fold difference	P^2^	Rate^3^	Fold difference	P^2^
Nonacanthomorphs	32	0.073			0.92		
Acanthomorphs	71	0.111	1.52	0.002	2.15	2.33	0.001

^1^ Disparity is estimated as Procrustes variance [31]

^2^ Determined from 1,000 permutations

^3^ Arbitrary units, based on Procrustes coordinates and divergence times [36,37]

Modeling with GEIGER [40, 41] yields a first-pass understanding of evolutionary dynamics that potentially could help to explain features of the morphospace patterning. As shown in Table 2, white noise, essentially a distribution not dependent on phylogeny, was not supported. The analysis also yielded only little support for “early burst” [43], a model characterizing ‘Simpsonian’ adaptive radiation in which rapid evolutionary changes occur approximately synchronously during the first stages of a radiation [42]. There is also little support for the “random walk” model of Brownian motion. Arguably, the Ornstein-Uhlenbeck model, positing adaptive directional evolution, is the best supported, considering PC1 and PC2 collectively.

**Table 2:** Tests of evolutionary models using the GEIGER platform, based on individual Principal Components.

	BM		WN		OU		EB	
Dataset	dAICc	dAICc SE	dAICc	dAICc SE	dAICc	dAICc SE	dAICc	dAICc SE
PC1	**0**	**0**	59.24	59.24	**0.23**	**0.27**	2.12	**1.33**
PC2	2.22	2.17	16.72	16.67	**0**	**0**	4.34	4.34

BM–Brownian Motion: evolution consistent with a random process.

WN–White Noise: essentially a nonphylogenetic model.

OU–Ornstein-Uhlenbeck: directional evolution toward an adaptive peak.

EB–Early Burst: an initial rapid and synchronous phase followed by more constrained change.

dAICc–delta AIC scores. dAICc_SE–delta AIC scores with correction for standard error. Generally, a dAIC score below 2 (bold-face) can be considered to be in support of the model, whereas scores above 2 (light-gray type face) suggest rejection of the model.

### Evolutionary convergences occur abundantly

We saw above that samples from different lineages can occupy closely neighboring positions on the morphospace landscape. For a striking example, consider the principal occupancy peak toward low PC1 and low PC2 on the landscape (i.e., to the lower left in Fig 3). Ten families are present above the highest contour line (Polypteridae, Lepisosteidae, Notopteridae, Ariidae, Osmeridae, Gobiidae, Atherinopsidae, Channichthyidae, Nototheniidae and Triglidae). The ten families include eight orders (sensu [13]) and nearly span the tree shown in Fig 4. This cohort, along with other examples, motivate us to entertain evolutionary convergence as a feature of the patterning of the landscape. We use convergence in the broad sense, to mean a pattern as defined by Stayton [41], and not to imply any underlying process such as adaptation.

**Fig 5:**
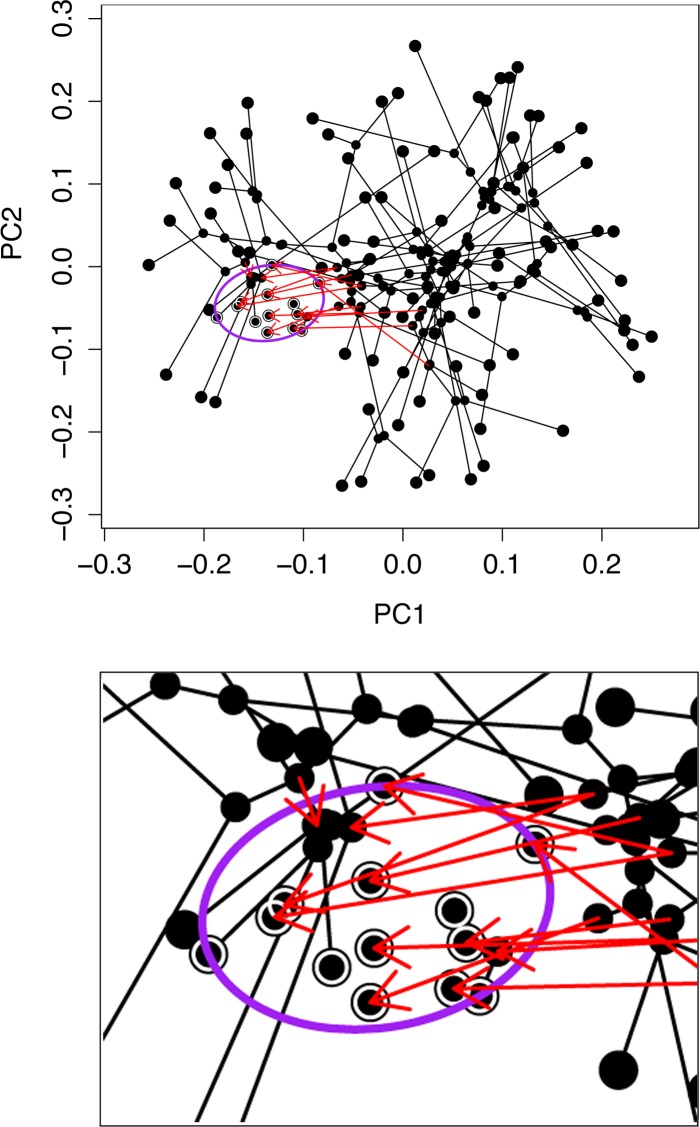
*Convevol* frequency-based measurement yields evidence for convergent evolution on the principal landscape occupancy peak (termed peak 1 in Table 3) of PC1 by PC2 phylomorphospace. The large points show terminal taxa (families) corresponding to points of the landscape plot shown in Fig 3. The smaller points show inferred positions of ancestors. Note the evolutionary trajectories, many of them quite long, that crisscross the landscape in a variety of directions. The ellipse (lower panel shows a zoom in) indicates the occupancy peak of interest, and the red arrows show independent entries (convergences) into this region. See text and Table 3 for further explanation.

We used the platform *convevol* [44] to directly examine whether convergence is a significant determinant of morphospace occupancy (Fig 5). The approach relies on the analysis of phylomorphospaces [11], which use morphological disparities, along with the phylogenetic connectivities and times of divergences of the branches of the phylogenetic tree to portray computed evolutionary pathways through morphospace. The positions of ancestors (branch points, indicated by smaller points in the Fig 5 plot) are calculated by squared-change parsimony, and the positions of the terminal taxa (larger points at the terminal ends of the branches in Fig 5) are empirical, matching the points of the Fig 3 landscape plot. The *convevol* output metric that we consider most important for our dataset, termed C_5_, describes the frequency of convergences into user-specified regions of a phylomorphospace, based on the computed positions of ancestors. The example in Fig 5 illustrates our findings. The phylomorphospace shows convergences onto the major landscape occupancy peak described above, here indicated by the lavender ellipse encompassing 13 families–the lower panel shows a zoomed-in view. *Convevol* detected 12 independent convergences into the region (red arrows), significant at an alpha level of 0.05, as determined from 1,000 simulations based on Brownian motion (P = 0.049).

**Fig 6:**
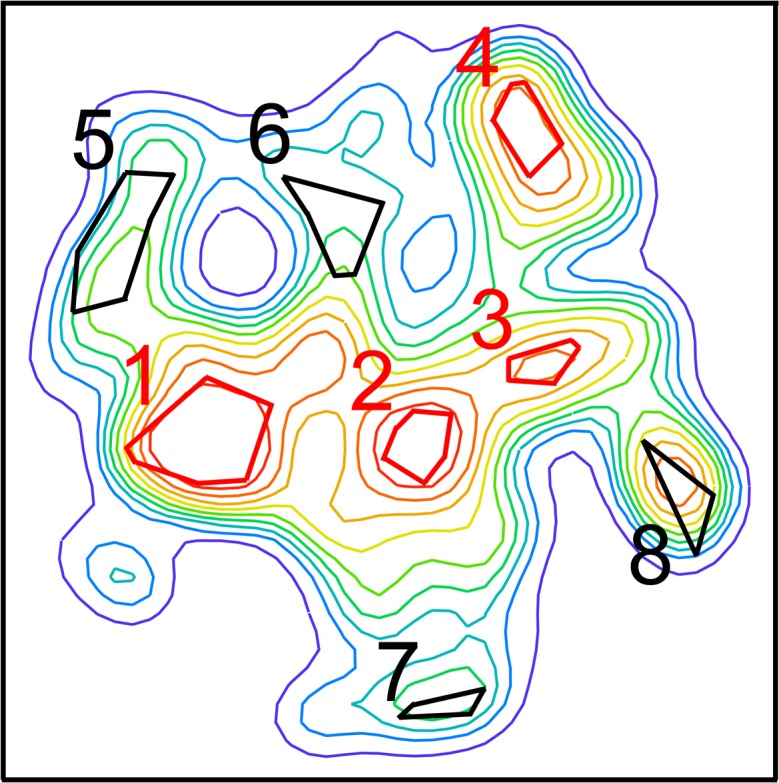
Key to the peak (in red) and off-peak (black) regions of the morphospace landscape analyzed by *convevol*, and reported in Table 3. Fig 3 shows the details of the morphospace.

These data are compared with seven similar analyses of the landscape, and examining not only the frequency-based measure C_5_, but also four similarity-based measures C_1_-C_4_ provided by *convevol* (Table 3; Fig 6 shows the analyzed locations on the landscape). C_1_, for example, quantifies the ratio of dissimilarity between terminal taxa to the maximal dissimilarity between any taxa in the two lineages; convergence is indicated where the terminal taxa are more similar. Of the eight regions analyzed, we selected four to represent the four prominent occupancy density peaks present on the landscape, and other four to represent off-peak regions (i.e., regions of lower elevations, such as the regions bordering the lowest elevation valleys; the very lowest troughs did not include enough datapoints for analysis). Peak 1, the example used in Fig 5, achieved significance for all of the metrics (C_1_ -C_5_), as did peak 2 and peak 4. Convergence onto peak 3 was only weakly supported, showing significance for only C_1_ and C_2_. Perhaps surprisingly, three of the four off-peak regions showed significance for all of the metrics, and the other, region 6, achieved significance for all of the metrics except C_5_. With six of the eight regions analyzed showing statistical support for all the metrics, and the other two showing partial support, *convevol* thus provides strong evidence for convergences occurring at multiple locations across the landscape.

**Table 3:** Frequency (C5) and similarity (C1-C4) based measures and probabilities (p) examining convergences within the indicated focal regions of the morphospace. *Convevol* analyses.

Metrics	Focal taxa *N*	C5		C1		C2		C3		C4	
*p* values			*p*(C5)		*p*(C1)		*p*(C2)		*p*(C3)		*p*(C4)
**Peak 1**	13	**12**	**0.049**	**0.74**	**0**	**0.148**	**0**	**0.012**	**0**	**0.012**	**0**
**2**	7	**8**	**0.09**	**0.709**	**0**	**0.108**	**0**	**0.009**	**0.002**	**0.009**	**0.003**
**3**	4	3	0.22	**0.518**	**0.006**	**0.054**	**0.011**	0.004	0.06	0.009	0.1
**4**	7	**7**	**0.025**	**0.763**	**0**	**0.154**	**0**	**0.012**	**0**	**0.017**	**0**
											
Off-peak 5	7	**7**	**0.044**	**0.535**	**0**	**0.085**	**0**	**0.007**	**0.002**	**0.007**	**0.002**
6	5	5	0.29	**0.674**	**0**	**0.123**	**0**	**0.01**	**0.001**	**0.011**	**0.001**
7	4	**4**	**0.007**	**0.851**	**0**	**0.246**	**0**	**0.019**	**0**	**0.02**	**0**
8	6	**6**	**0.026**	**0.708**	**0**	**0.155**	**0**	**0.012**	**0**	**0.18**	**0**

Peaks and off-peaks analyzed are identified in Fig 6. Probability (p) values determined from 1,000 simulations using Brownian motion. A probability of ‘0’ means that there were no values among the simulations as high as the empirical value of the metric. Highlighted cells show metrics significant at alpha = 0.05

### Both evolutionary convergence and divergence appear to contribute to morphospace patterning

In the preceding analyses, we examined particular small regions of the morphospace landscape for evidence of independent convergences among the neighbors occupying these regions. We can turn this approach around, and look at the morphological trajectories of OP shapes along lineages that are neighboring on the phylogenetic tree with respect to the entire morphospace. That is, what are the OP morphological differences among closely related families, and what does the phylomorphospace approach suggest about the nature of the divergences responsible for these differences?

**Fig 7:**
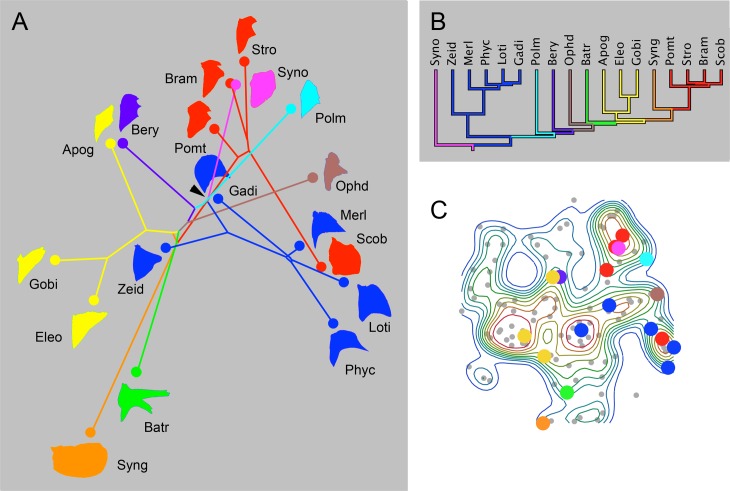
Phylomorphospace analyses reveals extensive evolutionary divergences, as well as apparent convergences among OP shapes in basal acanthomorphs. **A** The families included in the plot represent a contiguous paraphyletic grade of Eurypterygian fishes, as shown in the pruned tree in **B**. Fishes in the sister group to Scombromorpharia (sensu [13]) have been pruned from the tree. Colors facilitate tracing separate lineages, as explained in the text, across the landscape. **C** The landscape plot (points representing individual families as in Fig 3) shows the distribution of these families (color matched). For explanation of the abbreviations, see text and S1 Table.

To accomplish this analysis, we pruned small sets of neighboring branches out of the phylogenetic tree, with new color coding to aid in making out the details of the patterns of trajectories within these small sets. Exemplifying this approach, Fig 7A shows the phylomorphospace representing 18 families from the base of the neoteleosts. Fig 7B shows their phylogenetic relationships. Close relatives show loose clustering in the phylomorphospace (e.g., the blue clade encompassing four gadiform families and the sister group Zeidae). In contrast, the trajectories are not seemingly channeled, but scattered with respect to both direction and length. Syngnathidae (Syng) shows a particularly prominent long terminal trajectory on the phylomorphospace. The accompanying landscape plot (Fig 7C) reveals that the eighteen families of this clade are scattered across nearly the entire morphospace in the vertical dimension (representing PC2). Besides the divergences, apparent convergences are also suggested (e.g. Synodontidae (Syno) and Bramidae (Bram) converge toward one another at the top of the landscape).

**Fig 8:**
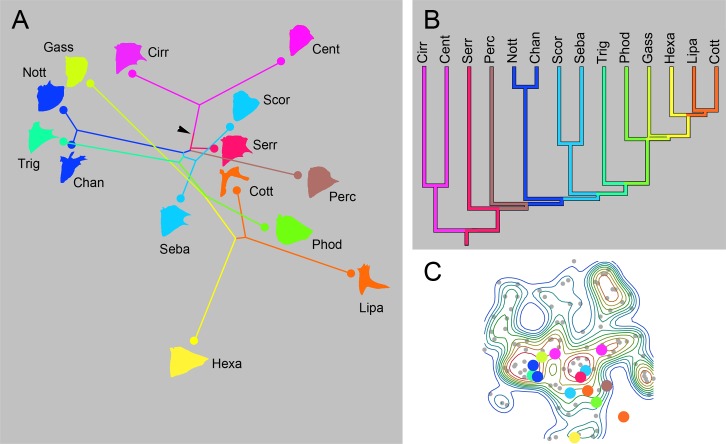
Phylomorphospace analysis of the ‘terminal’ (distal-most) clade of acanthomorphs, including Cottoidea and their allies. Presentation as in Fig 7. For explanation of the abbreviations, see text and S1 Table.

This pattern, suggesting both divergences and convergences, occur irrespective of what set of branches we analyze across the entire phylogenetic tree. We show another example that examines a perciform clade sampling the ‘bush at the top’ (as the collective of taxa in this terminal region of the phylogeny is frequently called) (Fig 8). As in Fig 7, we see as convergences–Nototheniidae (Nott), Channichthyidae (Chan) and Triglidae (Trig) onto occupancy peak 1, and Serranidae (Serr) and Scorpaenidae (Scor) onto peak 2. And we see, perhaps more prominently than convergences, long trajectories separating sister families, that are suggestive of marked divergences (Cirrhitidae (Cirr)–Centrarchidae (Cent) and Liparidae (Lipa)–Cottidae (Cott)).

We suggest from our phylomorphospace analyses that both divergence and convergence need to be considered as prominent mechanisms underlying landscape patterning and its ruggedness. We make this interpretation with caution, however, since using the phylomorphospace approach is not without errors, more substantial in the earlier stages of the lineages [11, 46].

## Discussion

### Teleost opercle macroevolution has produced a richly occupied morphospace

A principal finding of our study is that the local morphospace for the OP within teleosts is largely filled. Might dense occupancy be due to limited diversity, i.e., that OP shape is not evolving to any large extent? This potential explanation is not supported by our study: Our study set, while only representing about a fifth of teleost families, essentially spans the full phylogenetic tree of this numerous and richly variable group of vertebrates, although some groups such as deep-sea lineages are poorly represented. Visually comparing the bone shapes suggests the variation to be high. Furthermore, measurement of disparity using Procrustes variance suggests high disparity: For a rough comparison, we calculated OP morphological disparities using the Procrustes shapes of a single pair of oceanic and freshwater stickleback populations of three-spine stickleback from the study shown in S1 Fig: Our estimate for the microevolutionary stickleback disparities were 10-fold less than the values for our macroevolutionary dataset. To illustrate the difference directly on the morphospace examined in this study, S2 Fig (panel C) shows two points representing the disparity of the two stickleback morphs, as compared with the spread of the full species dataset.

Our finding of a well-filled morphospace in which clustering is not prominent is surprising, given the general understanding, since the pioneering work of Raup [3,4] and see [47], that morphospace occupancy is sparse [48,49,9]. In this model, occupied regions of the space, the ‘clumps’, are contained within substantial regions of unoccupied space. The lack of occupancy of the latter regions is presumed to be due to some kind of constraint on evolution. For example, lack of occupancy might be due to functional constraint–i.e., the morphologies that might have occupied the vacant regions are maladaptive [9,47]. Alternatively, developmental constraint could make morphospace regions inaccessible by the developmental process; [50,51,5]. The PCA-based empirical method that we examine in this study should have revealed a mostly empty morphospace if prominent constraints indeed were present: Examples of sparsely occupied morphospaces from our own work are the morphospaces describing OP development in zebrafish and stickleback. In both, variation is limited to a restricted zone of the space around nonlinear developmental trajectories, similar in form in both species. The trajectories completely avoid most of the space, including the central regions (Fig 5 in [15]; Fig 5 in [30]). Nevertheless, the unoccupied regions of the space we observe in the current study are not impressively large. In fact, they may result from our incomplete sampling of teleost taxa [9], and are not due to constraints.

An important dimension in the understanding of the evolution of our observed morphospace patterning is the addition of the phylogeny to its visualization–the phylomorphospace. A caveat is that phylomorphospaces are based on the positions of ancestors computed by squared change parsimony, not measurement of the morphologies of known ancestors. For this and other reasons the phylomorphospaces are likely to be only inaccurate portrayals of actual evolutionary changes (see also [11,46]). In spite of such problems, several examples in our study argue for both evolutionary convergences and divergences abundantly occurring within the morphospace. Both the convergences and divergences occur in a variety of directions, and frequently by long trajectories across the space. Both convergence and divergence have seemingly participated in a major way in filling the landscape.

### Developmental modularity could provide flexibility in shape evolution

Given the substantial variety of freshwater and marine habitats that different teleosts occupy, it seems likely that varying ecology and Darwinian adaptation have prominently influenced the patterning of the OP morphospace. Development might also play a role. In a study addressing opercle development in zebrafish we discovered that the different edges of the opercle grow at separate times from one another. Edge-specific growth is mediated by selective acquisition of early *sp7*-expressing osteoblasts [15]. A mutational study showed that the osteoblasts along one of these edges specifically, use a Hedgehog signal to stimulate cell division in an adjacent pre-osteoblast population. The signal thus provides regulation of the size of the pre-osteoblast pool, and indirectly, the rate of local growth of the bone [16]. These observations suggest that patterning morphology of the bone is modular, where developmental modules are defined as semiautonomous units of morphological patterning [52–54]. In this case, we proposed that modularity is controlling time- and position-specific features of local bone formation and growth–and hence, region by region, the shape of the opercle [15,16,55].

We extended the zebrafish studies to sticklebacks, comparing development in ancestral (oceanic) and derived (freshwater) populations, and showed that opercle evolution in this species involves a kind of “dissociated heterochrony” [56] in which at one developmental stage, a single region of the bone develops at different rates in the two forms. Strikingly, examining the opercle’s covariance structure by Klingenberg’s method [57] provided direct evidence for modularity, and found that the evolutionary change in bone shape is along a module boundary, the location of the boundary reasonably well predicted from the zebrafish work [30].

This previous work, with single species, informs macroevolution [55]. Modules, as semiautonomous units, would allow for regional dissociability, providing evolutionary ‘flexibility’ among species and higher taxa, and potentially channeling evolution in different directions in different taxa. We propose that the rich morphospace occupancy and the diverse trajectories through the space that we observe depend in part on modular development. As we better understand the developmental genetic underpinnings of these modules, one hope is that future comparative work leveraging detailed genomic information from a host of teleost species will help explain how module tinkering contributes to biodiversity over evolutionary time.

### An evaluation of possible constraints on OP shape

**Fig 9:**
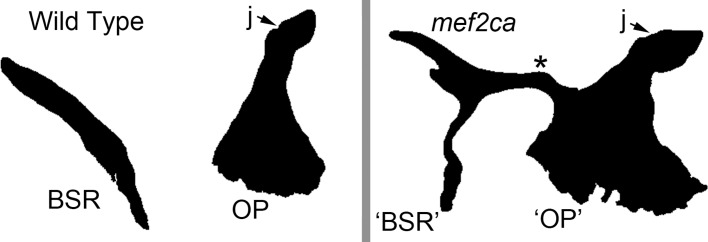
In this zebrafish *mef2ca* mutant a bridge of bone (*) forms between the OP anterior-ventral bone and a branchiostegal ray (BSR). Early larva stage–mutants die soon after this time (examples from [17]; see also [18]).

That the OP is invariably a flat bone, and invariably forms the socket for the ball-and-socket joint with the hyomandibula shows constraint on morphology. One can easily imagine that these are functional constraints, given that the bone supports a flattened operculum, the movement of which mediates gill-ventilation. The OP also makes close associations with other bones, notably the subopercle, but in spite of this potentially constraining influence, we observed considerable shape variation of this OP edge, the posterior-ventral edge where it articulates with the subopercle. In contrast, we noted in the Results section the apparently low shape variation of the anterior-ventral edge of the OP. This edge abuts the preopercle, connecting with it via a ligament, and movement across the edge occurs during respiration. Accordingly, one might suppose that shape conservation represents functional constraint. However, from our zebrafish study, this OP edge is seemingly an early module in itself [14]; and one might alternatively suppose that variability of the module and edge shape is constrained developmentally, rather than functionally. There exist several routes toward attempting to find out whether a putative constraint is developmental or otherwise [9, 58]. In what he termed “the logic of monsters”, Alberch [58] points out that one might observe a maladaptive and strange phenotype in artificial situations, and proving that the phenotype is possible developmentally even though not observed in nature (see also [9]). In analyses of zebrafish homozygous for a lethal mutant allele of the transcription factor-encoding gene *mef2ca* we find that the anterior ventral edge of the OP can re-shape dramatically (Fig 9). By the logic of monsters argument, this finding suggests that developmental constraint is not the best explanation for why this edge is conservative among naturally occurring variants.

### Dynamic evolution of the opercle in acanthomorphs

Adaptation might be the principal driver of opercle morphology. We observed prominent occupancy peaks across the landscapes which could be due to adaption, but we note that equating adaptive and occupancy peaks requires additional supporting evidence [47,59]. Our finding multiple convergences with the *convevol* platform might also suggest adaptation, but this interpretation also is not compelling, since convergences could be due to constraints, or even to neutral processes [45]. Our GEIGER analysis may be relevant to this issue in that an adaptive model, Ornstein-Uhlenbeck, is supported using each of the leading two principal components of OP shape in our trait analysis.

Evolution of opercle shapes in acanthomorphs may have been especially dynamic. The acanthomorph OPs spread more than other groups across the landscape, and the phylomorphospace plots suggest prominent trajectories occur throughout. Previous studies of body morphology [26,27] see also [60] demonstrate that acanthomorphs have opportunistically and ‘explosively’ occupied morphospace newly vacated by the end-Cretaceous extinction event. Our results seem to complement these recent paleontological and comparative findings. Further, we wonder whether, in addition to the body forms studied by Friedman and associates, our results could mean that explosive morphological evolution within the acanthomorphs extends to head bone shapes. Further study of skull bone morphologies, particularly as related to the ecology of lineages of acanthomorph fish could address this question.

## Supporting information

S1 Table Families and exemplar species studied. Branch number refers the terminal branches of the pruned phylogenetic tree [13]. 0 indicates the family was not included in the Betancur-R study.
(DOCX)

S1 Fig Morphospace showing microevolution in stickleback. Global microevolutionary OP divergences in threespine stickleback obtained from principal component analysis (PCA) yield a well-filled local morphospace. Red filled circles: oceanic (ancestral form, 8 populations). Black open circles, freshwater (derived forms, 14 populations. Notice that within the single cluster of OP forms, the two morphs are mostly separated along PC1. The configurations (right) show the deformation along PC1 (gray: oceanic, black: freshwater, arrows indicating prominent shape change). The plot is original, the data (available from Dryad) are from [21].
(TIF)

S2 Fig **Comparability of OP datasets A-C), and examples of within-family variation (D).** Morphospaces as in Fig 3 of the main text. **A** The same landmark-based data with sample points repeating the presentation Fig 3 of the main text. **B** shows the same samples, but with a PCA morphospace using an outline method based on Elliptical Fourier transformation. The outlines were captured by digitizing 400 xy points for each sample, moved to *PAST* [Hammer Ø, Harper DAT, Ryan PD. PAST: Paleontological Statistics Software Package for Education and Data Analysis. Palaeontol. Electronica 2001;4: 9pp], and transformed into 2D shape coordinates including 30 modes (harmonics). Size information is removed by this procedure. Silhouettes in A and B show the sample shapes and colors show phylogenetic relationships, matching Fig 3 and the branches of the tree in Fig 4. The individual OPs generally map to approximately the same regions of both spaces, such that samples near one another on one of the spaces, are also usually also near one another on the other. Whereas the elliptical Fourier and landmarking methods are clearly revealing different aspects of Op shape, there is also some consistency between the two kinds of space-shape mappings. **C** Including more species in PCA morphospace using landmarking does not yield a substantially more clustered distribution of samples. Rather the space is more densely occupied, as compared with the plot made from only single species per family (A). The two larger points show overlays representing oceanic (filled, black circle) and lake (open circle) stickleback, described in the Discussion. The configuration diagrams indicate shape changes associated with the PC axes (consensus shape in light blue in the overlays). **D** The same morphospace as C, but with the landmarked species of five families highlighted to show examples of within-family shape variation, as compared with among-family variation represented by the whole plot. Colors match the other panels. Cichlidae (Cich) shows the most extreme within-family variation encountered in this study. The others are more typical: Characidae (Char), Salmonidae (Salm), Labridae (Labr), Scombridae (Scob).
(TIF)
