# Supplementary material for: A rich diversity of opercle bone shape among teleost fishes
Source: PLoS One. 2017 Dec 27;12(12):e0188888. doi: 10.1371/journal.pone.0188888 (PMC5744915; doi:10.1371/journal.pone.0188888)
Supplement: S1 Table — Branch number refers the terminal branches of the pruned phylogenetic tree [13]. 0 indicates the family was not included in the Betancur-R study. (DOCX) [file pone.0188888.s001.docx]

| **Branch #** | **Family abbreviation** | **Family** | **Exemplar:**  ***Genus*** | ***species*** | **Specimen**  **catalog #** |
| --- | --- | --- | --- | --- | --- |
| 87 | Acan | Acanthuridae | *Zebrastoma* | *flavescens* | m077 |
| 67 | Adri | Adrianichthyidae | *Oryzias* | *latipes* | m338 |
| 16 | Ales | Alestiidae | *Phenacogrammus* | *interruptus* | m202 |
| 2 | Amii | Amiidae | *Amia* | *calva* | m094 |
| 74 | Ammo | Ammodytidae | *Ammodytes* | *hexapterus* | m298 |
| 54 | Anab | Anabantidae | *Ctenopoma* | *ansorgii* | m248 |
| 4 | Angu | Anguillidae | *Anguilla* | *rostrata* | m267 |
| 0 | Anos | Anostomidae | *Anostomus* | *anostomus* | m074 |
| 42 | Apog | Apogonidae | *Pterapogon* | *kauderni* | m045 |
| 14 | Apte | Apteronotidae | *Aperonotus* | *leptorhynchus* | m277 |
| 24 | Arii | Ariidae | *Ariopsis* | *seemanni* | m260 |
| 0 | Aspr | Aspredinidae | *Bunocephalus* | *coracoideus* | m056 |
| 62 | Athe | Atherinopsidae | *Menidia* | *menidia* | m296 |
| 85 | Bals | Balistidae | *Rhinecanthus* | *rectangulus* | m233 |
| 0 | Balt | Balitoridae | *Beaufortia* | *kweichowensis* | m022 |
| 41 | Batr | Batrachoididae | *Opsanus* | *tau* | m090 |
| 39 | Bery | Berycidae | *Beryx* | *decadactylus* | m310 |
| 73 | Blen | Blenniidae | *Ecsenius* | *bicolor* | m042 |
| 48 | Bram | Bramidae | *Brama* | *australis* | m284 |
| 22 | Call | Callichthyidae | *Corydoras* | *aeneus* | m020 |
| 81 | Capr | Caproidae | *Antigonia* | *capros* | m315 |
| 57 | Cara | Carangidae | *Caranx* | *crysos* | m271 |
| 11 | Cato | Catostomidae | *Catostomus* | *sp* | m002 |
| 91 | Cent | Centrarchidae | *Micropterus* | *salmoides* | m276 |
| 88 | Chae | Chaetodontidae | *Chaetondon* | *ocellatus* | m068 |
| 95 | Chan | Channichthyidae | *Champsocephalsus* | *gunnari* | m381 |
| 20 | Char | Characidae | *Hyphessobrycon* | *166* | m166 |
| 61 | Cich | Cichlidae | *Tilapia* | *sp* | m008 |
| 90 | Cirr | Cirrhitidae | *Neocirrhites* | *armatus* | m173 |
| 10 | Clup | Clupeidae | *Alosa* | *sapidissima* | m157 |
| 12 | Cobi | Cobitidae | *Chromobotia* | *macracanthus* | m185 |
| 5 | Cong | Congridae | *Conger* | *oceanicus* | m313 |
| 102 | Cott | Cottidae | *Cottus* | *rhotheus* | m331 |
| 65 | Cypd | Cyprinodontidae | *Cyprinodon* | *salinus* | m321 |
| 13 | Cypn | Cyprinidae | *Cyprinus* | *carpio* | m001 |
| 27 | Dora | Doradidae | *Oxydoras* | *niger* | m076 |
| 43 | Eleo | Eleotridae | *Valenciennea* | *puellaris* | m073 |
| 69 | Embi | Embiotocidae | *Amphistichus* | *rhodoterus* | m163 |
| 9 | Engr | Engraulidae | *Engraulis* | *mordax* | m108 |
| 78 | Ephi | Ephippidae | *Platax* | *batavianus* | m054 |
| 29 | Esoc | Esocidae | *Esox* | *lucius* | m007 |
| 66 | Fund | Fundulidae | *Fundulus* | *heteroclitus* | m142 |
| 37 | Gadi | Gadidae | *Melanogrammus* | *aeglefinus* | m132 |
| 19 | Gass | Gasteropelecidae | *Carnegiella* | *strigata* | m250 |
| 100 | Gass | Gasterosteidae | *Gasterosteus* | *aculeatus* | m113 |
| 44 | Gobi | Gobiidae | *Gobiodon* | *okinawae* | m174 |
| 72 | Gobs | Gobiesocidae | *Gobiesox* | *meandricus* | m361 |
| 0 | Grac | Grammicolepididae | *Xenolepidichthys* | *dalgleishi* | m303 |
| 71 | Grat | Grammatidae | *Gramma* | *loreto* | m043 |
| 53 | Helo | Helostomatidae | *Helostoma* | *temminckii* | m211 |
| 0 | Hemt | Hemitripteridae | *Hemitript* | *cirrhosus* | m330 |
| 101 | Hexa | Hexagrammidae | *Ophiodon* | *elongatus* | m096 |
| 7 | Hiod | Hiodontidae | *Hiodon* | *alosoides* | m358 |
| 0 | Hypo | Hypopomidae | *Brachyhypopomus* | *pinnicaudatus* | m279 |
| 23 | Icta | Ictaluridae | *Ictalurus* | *punctatus* | m005 |
| 50 | Indo | Indostomidae | *Indostomus* | *paradoxus* | m323 |
| 56 | Istio | Istiophoridae | *Tetrapturus* | *audax* | m383 |
| 76 | Labr | Labridae | *Tautoga* | *onitis* | m273 |
| 18 | Lebi | Lebiasinidae | *Nannostomus* | *mortenthaleri* | m191 |
| 3 | Lepi | Lepisosteidae | *Lepisosteus* | *osseus* | m167 |
| 103 | Lipa | Liparidae | *Liparis* | *florae* | m360 |
| 82 | Loph | Lophiidae | *Lophius* | *americanus* | m089 |
| 21 | Lori | Loricariidae | *Hypostomus* | *plecostomus* | m182 |
| 36 | Loti | Lotidae | *Lota* | *lota* | m154 |
| 51 | Mast | Mastacembelidae | *Macrognathus* | *siamensis (?)* | m240 |
| 63 | Mela | Melanotaeniidae | *Melanotaenia* | *praecox* | m223 |
| 34 | Merl | Merlucciidae | *Merluccius* | *gayi* | m286 |
| 25 | Moch | Mochokidae | *Synodontis* | *batensoda ?* | m023 |
| 77 | Moro | Moronidae | *Morone* | *saxatilis* | m003 |
| 8 | Notp | Notopteridae | *Chitala* | *ornata* | m192 |
| 94 | Nott | Nototheniidae | *Gobionotothen* | *gibberifrons* | m379 |
| 83 | Ogco | Ogcocephalidae | *Dibranchus* | *atlanticus* | m314 |
| 6 | Ophc | Ophichthidae | *Ophichthus* | *cruentifer* | m307 |
| 40 | Ophd | Ophidiidae | *Genypterus* | *maculatus* | m292 |
| 31 | Osme | Osmeridae | *Osmerus* | *mordax* | m111 |
| 52 | Osph | Osphronemidae | *Trichogaster* | *trichopterus* | m205 |
| 58 | Para | Paralichthyidae | *Paralichthys* | *dentatus* | m124 |
| 93 | Perc | Percidae | *Perca* | *flavescens* | m371 |
| 99 | Phod | Pholidae | *Pholis* | *gunnellus* | m306 |
| 60 | Phot | Pholidichthyidae | *Pholidichthys* | *leucotaenia* | m190 |
| 35 | Phyc | Phycidae | *Urophycis* | *regia* | m129 |
| 28 | Pime | Pimelodidae | *Pseudoplatystoma* | *sp* | m062 |
| 75 | Ping | Pinguipedidae | *Prolatilus* | *jugularis* | m290 |
| 59 | Pleu | Pleuronectidae | *Hippoglossus* | *stenolepis* | m156 |
| 64 | Poec | Poeciliidae | *Poecilia* | *reticulata* | m032 |
| 38 | Polm | Polymixiidae | *Polymixia* | *lowei* | m309 |
| 1 | Polp | Polypteridae | *Polypterus* | *bichir* | m254 |
| 89 | Poma | Pomacanthidae | *Pygoplites* | *diacanthus* | m037 |
| 70 | Pome | Pomacentridae | *Amphiprion* | *clarkii* | m067 |
| 46 | Pomt | Pomatomidae | *Pomatomus* | *saltatrix* | m084 |
| 17 | Proc | Prochilodontidae | *Semaprochilodus* | *taniurus* | m198 |
| 0 | Rivu | Rivulidae | *Austrofundulus* | *limnaeus* | m320 |
| 30 | Salm | Salmonidae | *Oncorhynchus* | *nerka* | m283 |
| 86 | Scia | Sciaenidae | *Leiostomus* | *xanthurus* | m270 |
| 49 | Scob | Scombridae | *Thunnus* | *albacares* | m384 |
| 96 | Scor | Scorpaenidae | *Dendrochirus* | *zebra* | m075 |
| 68 | Scos | Scomberesocidae | *Scomberesox* | *saurus* | m311 |
| 97 | Seba | Sebastidae | *Sebastes* | *maliger* | m099 |
| 92 | Serr | Serranidae | *Centropristis* | *striata* | m125 |
| 80 | Siga | Siganidae | *Siganus* | *vulpinus* | m177 |
| 26 | Silu | Siluridae | *Kryptopterus* | *bicirrhis* | m216 |
| 79 | Spar | Sparidae | *Stenotomus* | *chrysops* | m082 |
| 55 | Sphy | Sphyraenidae | *Sphyraena* | *argentea* | m102 |
| 15 | Ster | Sternopygidae | *Sternopygus* | *macrurus* | m278 |
| 47 | Stro | Stromateidae | *Peprilus* | *triacanthus* | m263 |
| 45 | Syng | Syngnathidae | *Syngnathus* | *fuscus* | m305 |
| 32 | Syno | Synodontidae | *Trachinocephalus* | *myops* | m345 |
| 84 | Tetra | Tetraodontidae | *Tetraodon* | *sp* | m057 |
| 98 | Trig | Triglidae | *Aspitrigla* | *cuculus* | m087 |
| 33 | Zeid | Zeidae | *Zenopsis* | *conchifera* | m316 |
|  |  |  |  |  |  |
