# Supplementary figures and images for: A rich diversity of opercle bone shape among teleost fishes
Source: PLoS One. 2017 Dec 27;12(12):e0188888. doi: 10.1371/journal.pone.0188888 (PMC5744915; doi:10.1371/journal.pone.0188888)

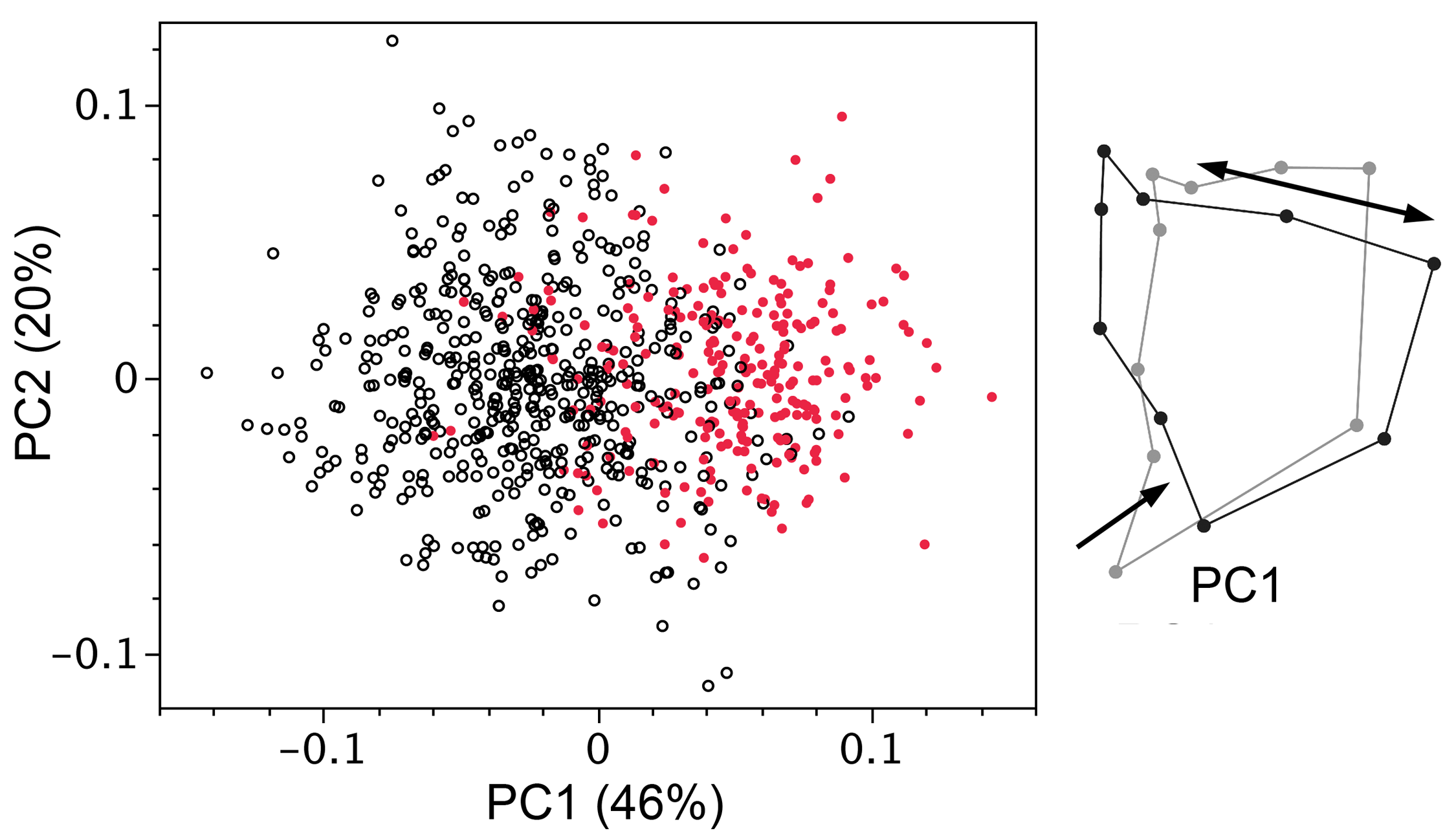

Supplement: S1 Fig — Global microevolutionary OP divergences in threespine stickleback obtained from principal component analysis (PCA) yield a well-filled local morphospace. Red filled circles: oceanic (ancestral form, 8 populations). Black open circles, freshwater (derived forms, 14 populations. Notice that within the single cluster of OP forms, the two morphs are mostly separated along PC1. The configurations (right) show the deformation along PC1 (gray: oceanic, black: freshwater, arrows indicating prominent shape change). The plot is original, the data (available from Dryad) are from [21]. (TIF) [file pone.0188888.s002.tif]

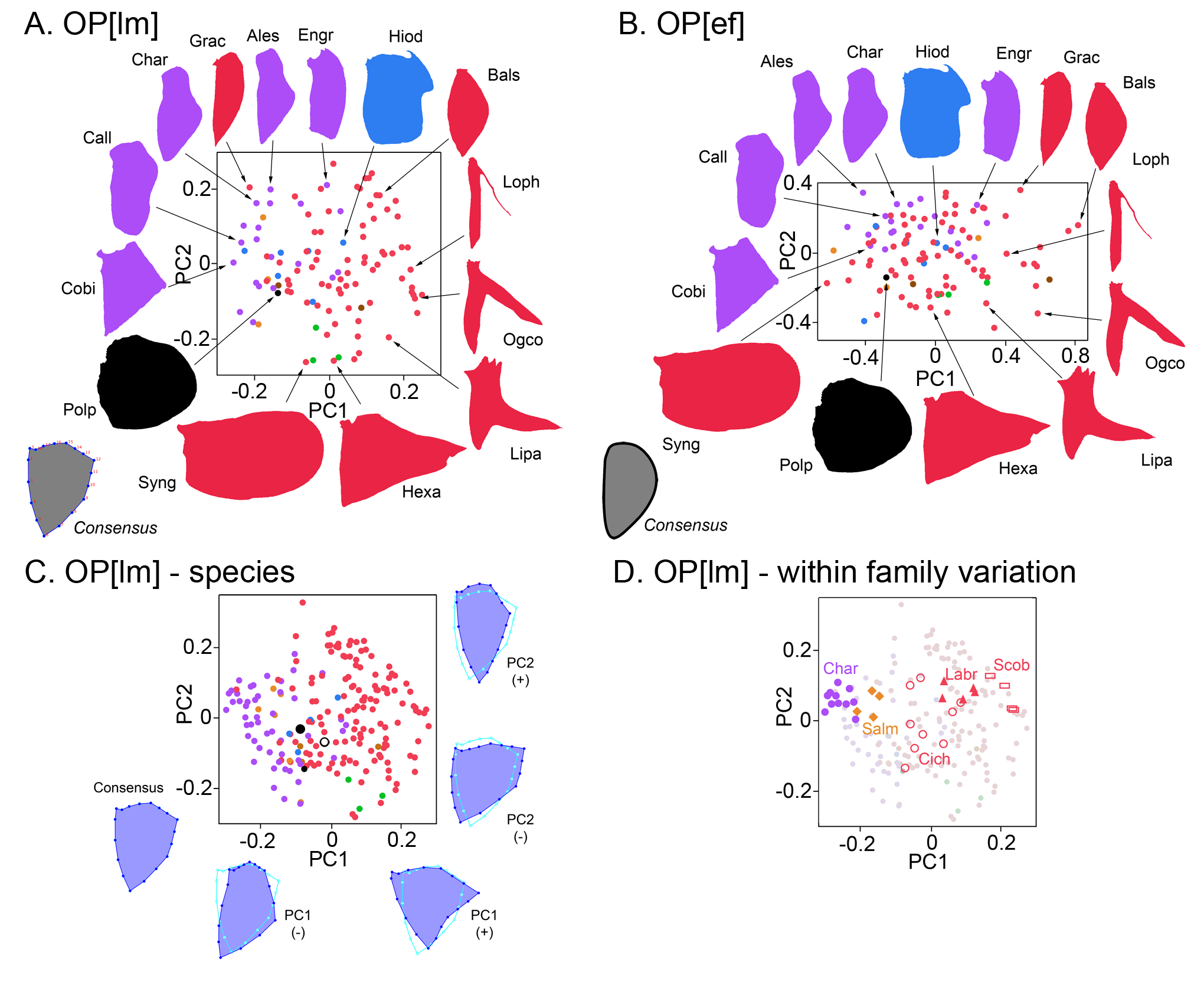

Supplement: S2 Fig — Comparability of OP datasets A-C), and examples of within-family variation (D). Morphospaces as in Fig 3 of the main text. A The same landmark-based data with sample points repeating the presentation Fig 3 of the main text. B shows the same samples, but with a PCA morphospace using an outline method based on Elliptical Fourier transformation. The outlines were captured by digitizing 400 xy points for each sample, moved to PAST [Hammer Ø, Harper DAT, Ryan PD. PAST: Paleontological Statistics Software Package for Education and Data Analysis. Palaeontol. Electronica 2001;4: 9pp], and transformed into 2D shape coordinates including 30 modes (harmonics). Size information is removed by this procedure. Silhouettes in A and B show the sample shapes and colors show phylogenetic relationships, matching Fig 3 and the branches of the tree in Fig 4. The individual OPs generally map to approximately the same regions of both spaces, such that samples near one another on one of the spaces, are also usually also near one another on the other. Whereas the elliptical Fourier and landmarking methods are clearly revealing different aspects of Op shape, there is also some consistency between the two kinds of space-shape mappings. C Including more species in PCA morphospace using landmarking does not yield a substantially more clustered distribution of samples. Rather the space is more densely occupied, as compared with the plot made from only single species per family (A). The two larger points show overlays representing oceanic (filled, black circle) and lake (open circle) stickleback, described in the Discussion. The configuration diagrams indicate shape changes associated with the PC axes (consensus shape in light blue in the overlays). D The same morphospace as C, but with the landmarked species of five families highlighted to show examples of within-family shape variation, as compared with among-family variation represented by the whole plot. Colors match the other panels. Cichlidae [file pone.0188888.s003.tif]
